# The Chronic Care for Wet Age Related Macular Degeneration (CHARMED) Study: A Randomized Controlled Trial

**DOI:** 10.1371/journal.pone.0143085

**Published:** 2015-11-16

**Authors:** Stefan Markun, Avraham Dishy, Stefan Neuner-Jehle, Thomas Rosemann, Anja Frei

**Affiliations:** 1 Institute of Primary Care, University Hospital of Zurich, University of Zurich, Zurich, Switzerland; 2 Department of Ophthalmology, Cantonal Hospital Aarau, Aarau, Switzerland; 3 Institute of Social and Preventive Medicine, University of Zurich, Zurich, Switzerland; University of Florence, ITALY

## Abstract

**Background:**

In real life, outcomes in wet age related macular degeneration (W-AMD) continue to fall behind the results from randomized controlled trials. The aim of this trial was to assess if outcomes can be improved by an intervention in healthcare organization following recommendations of the Chronic Care Model (CCM).

**Methods:**

Multi-centered randomized controlled clinical trial. The multifaceted intervention consisted in reorganization of care (delivery by trained chronic care coaches, using reminder systems, performing structured follow-up, empowering patients in self-monitoring and giving decision-support). In the control usual care was continued. Main outcome measures were changes in ETDRS visual acuity, optical coherence tomography (OCT) macular retinal thickness and quality of life (NEI VFQ-25 questionnaire).

**Results:**

169 consecutive patients in Swiss ophthalmology centers were included. Mean ETDRS baseline visual acuity of eyes with W-AMD was 57.8 (± 18.7). After 12 months, the between-group difference in mean change of ETDRS visual acuity was -4.8 (95%CI: -10.8 to +1.2, p = 0.15); difference in mean change of OCT was +14.0 (95% CI -39.6 to 67.6, p = 0.60); difference in mean change of NEI VFQ-25 composite score mean change was +2.1(95%CI: -1.3 to +5.5, p = 0.19).

**Conclusions:**

The intervention aiming at improving chronic care was not associated with favorable outcomes within 12 months. Other approaches need to be tested to close the evidence-performance gap in W-AMD.

**Trial Registration:**

Controlled-Trials.com ISRCTN32507927

## Introduction

Wet age related macular degeneration (W-AMD) is a chronic and progressive disease leading to loss of visual acuity and is already the most common cause for acquired blindness in developed countries.[1, 2] Choroidal neovascularizations are the pathological process leading to visual loss in W-AMD. Since the development of monoclonal antibodies and inhibitors targeting the vascular endothelial growth factor A (Ranibizumab, Bevacizumab and Aflibercept), effective treatments applied by intravitreal injections are available.[3] The MARINA, ANCHOR and the VIEW1 and 2 studies are regarded as the cornerstone studies in the use the Anti VEGF-A agents in W-AMD and demonstrated that the decline of visual acuity caused by W-AMD can not only be delayed or halted but even reversed and clinically relevant gains of visual acuity can be achieved.[4–6]

Interestingly, in real life outcomes are found to be less favorable than in clinical trials.[7] The most plausible explanation for this finding an evidence-performance gap (e.g. patients in real life do not receive care as provided in interventions within clinical trials). In real life, healthcare for AMD is challenging, not only because of the considerable treatment costs and because of the controversies about the different propagated treatment-regimes but also because of disease inherent reasons. The natural course of W-AMD is insidious because active and inactive stages alternate unpredictably and with considerable variability between patients. Is has been shown that treatment discontinuation because of disease inactivity is associated with considerable visual loss within only 4 years.[8] The importance of successful clinical follow-up is undoubted, independently whether a fixed dosage regime as labelled is followed or whether W-AMD is managed with the popular “pro re nata” or the “treat and extend” protocols.[9, 10] Furthermore, the time from symptom begin to treatment initiation is paramount. Treatment effects from anti VEGF-A drugs are only prominent when treatment is initiated within the first month of symptom onset.[11] Congruently, guidelines not only recommend regular follow-up visits but also encourage self-monitoring by patients i.e. by applying self-administered Amsler tests.[12]

Successful follow-up and patient empowerment e.g. in self-monitoring are recognized in treatment of AMD as they are in most classic chronic conditions such as hypertension, diabetes and many more. However, there are more relevant aspects of chronic illness care and all these have been encompassed comprehensively by Wagner and colleagues within the “Chronic Care Model” (CCM). The CCM is a generic, evidence-based template designed as a conceptual framework integrating the relevant elements of chronic care that have to be considered to achieve best possible outcomes in chronic conditions.[13] In addition to follow-up management and patient-empowerment, the CCM aims at mobilization of community resources, tailoring of the delivery system design, enhance decision-support and integrate clinical information systems. Interventions targeting at least two of these elements have consistently shown to improve outcomes in several typical chronic conditions such as diabetes, chronic obstructive pulmonary disease (COPD), asthma or depression as systematic reviews confirm.[14–17]

The CCM has widespread acceptance and is even promoted by the WHO as the ideal template to provide care in chronic conditions.[18] The CCM can be applied also in W-AMD and elements such as empowerment of patients in self-monitoring for example have already shown to enhance specific outcomes in W-AMD.[19] The CCM has however, not yet been tested in a broader implementation as a whole framework in care for W-AMD regarding patient-relevant outcomes.

We hypothesized that the implementation of the CCM might fill the observed evidence-performance gap and improve outcomes in W-AMD. We therefore undertook a multicenter randomized clinical trial aiming to improve visual function (primary objective) and quality of live (secondary objective) by implementing core elements of the CCM in care for W-AMD.

## Methods

### Study design

The CHARMED study was a multicenter, open label, randomized, controlled clinical trial that was conducted in 12 different Swiss ophthalmology clinics. The aim of the CHARMED study was to assess the effects of an implementation of the CCM in care for patients with W-AMD. The methods of the study have been reported in detail and are summarized below.[20]

Recruiting of consecutive patients in the study centers started in April 2011 and ended in January 2013. Randomization was performed on the patient level utilizing block randomization stratified per study center, block size was 4 and allocation ratio was 1:1. The randomization list was generated using STATA statistical software version 11, the list was stored centrally at the Institute for Primary Care. Study centers had no access to this list and had to inquire the allocation of each individual patient by telephone call immediately after patient inclusion.

Eligible subjects were male or female patients, aged 50 years or older, with W-AMD, who were eligible for therapy with antiangiogenic drugs, had a best corrected visual acuity of at least 20 letters assessed with the Early Treatment Diabetic Retinopathy (ETDRS) chart and provided written informed consent in study participation. In cases where both eyes were affected by W-AMD, both eyes were included and followed in the study.

Exclusion criteria were serious general or psychological illness (advanced malignant diseases, severe depressive disorders or dementia) and insufficient German or French language skills (for completing the self-administered questionnaires). We intended to include treatment-naïve patients, thus patients with any former invasive medical treatment for W-AMD were also excluded.

Data was collected at inclusion (baseline, T0), at 6 months follow-up (T1) and at 12 months follow-up (T2). Clinical measurements (OCT and ETDRS visual acuity) were conducted by ophthalmologists who received instructions in standardized measurements using the ETDRS chart, structured interviews were conducted by trained practice assistants in a face-to-face or telephone setting and self-administered questionnaires were completed by the patients.

### Interventions

In the control group, there was no study specific intervention. The study sites were instructed to deliver care as usual and to avoid introduction of intervention-specific components.

In the intervention group, established evidence based core elements of the CCM were introduced. Delivery of these CCM elements was organized as follows: In every study site a practice assistant was assigned to be the “Chronic Care Coach” (CCC). The CCCs attended a one-day training course comprising the instructions and materials to utilize as means to introduce the CCM core elements. In specific the following elements were introduced:

#### Organization of health care delivery system

In the intervention group, care was organized by the CCCs. This included planning and arranging the contact between patients and physicians and a monthly structured follow-up call with the patients. The CCCs used, delivered or implemented the diverse elements or instruments of the multifaceted intervention.

#### Self-management support

Patients in the intervention group were individually instructed in self-management by the CCCs. These private teaching sessions comprised the self-assessment of the Amsler-test, which was to be performed by the patient weekly with both eyes separately. Furthermore, patients in the intervention group received an action plan that helped them dealing with symptoms, estimate their severity and how to react in the case of deterioration. The action plan also contained an overview of the planned visits and the contact information of the CCC. Additionally, to enhance self-management support, patients in the intervention group were invited to peer-group meetings with experienced patients suffering from W-AMD at least twice during the 12 month intervention period. Originally we also planned to introduce self-measuring on the patient level utilizing an app designed to monitor visual acuity on a mobile device, this component, however, was abandoned as the visual acuity test on the app failed to demonstrate validity according to the manufacturer.

#### Decision support

Patients in the intervention group were provided with leaflets by the CCCs containing “Don’t worry” and “Call immediately” items including a checklist for antibiotic eye drops and all important contact addresses.

#### Clinical information systems

A computer system designed to remind and structure the follow-up of patients in the intervention group was used by the CCCs. The system targeted at achieving three monthly injections at study begin by promptly fixing these appointments at treatment start, and aimed at successful monthly visits thereafter. The monthly phone calls were also reminded and managed with this computer system.

### Outcome measures

Measurements were performed at baseline (T0), at 6 months (T1) and at 12 months (T2). The points in time where the individual outcome measurements were performed are reported below:

#### Primary outcome

The primary outcome measure was the best corrected visual acuity measured with the ETDRS chart in sitting position from an initial test distance of 4 meters at T0, T1 and T2. The primary outcome was the mean change in the ETDRS after 12 months. A change of 5 letters was regarded as minimal clinically relevant change. In order to enhance standardization of this measurement an initial teaching visit and at least one outreach visit at each study center was performed by a study coordinator. Standardized ETDRS charts were provided.

#### Secondary outcomes

1) The physiological outcome was assessed by optical coherence tomography (OCT) of the central retinal thickness at T0, T1 and T2. 2) The disease-specific quality of life assessed with the National Eye Institute Visual Function Questionnaire-25 (NEI VFQ-25).[21] The NEI VFQ-25 was performed at T0, T1 and T2 by face-to-face or telephone interview. 3) Health service utilization was assessed by self-administered questionnaire (counting the number of visits with the ophthalmologists, with the family physician and stays in hospitals) at T0 and T2. 4) The patient perceived accordance of care to the CCM was measured by the self-administered Patient Assessment of Chronic Illness Care (PACIC) questionnaire at T0 and T2.[22] 5) The provider perspective on the accordance of care to the CCM was assessed using the Assessment of Chronic Illness Care (ACIC) questionnaire which was to be completed in collaboration by the clinic staff at the beginning of recruitment and after follow-up was completed.[23]

### Sample Size

According to literature, we assumed a mean EDTRS visual acuity of 48 letters and a standard deviation (SD) of 15 letters at baseline.[5, 24, 25] Assuming a clinically relevant between-group difference of 5 letters on the ETDRS chart, we needed to include 282 patients to achieve a power of 80% and a significance level of 5%. The CHARMED–Study-Group, however, decided to stop recruitment after 169 patients, because the recruitment proceeded much slower than expected.

### Trial registration and ethics statement

The trial has been registered at Current Controlled Trials (ISRCTN32507927). Ethics committee approval was obtained (Ethics Committee of the Canton of Zurich, KEK-ZH-NR: 2010-04391/1). Informed consent was obtained from subjects prior to inclusion, the study was performed adhering to the tenets of the Declaration of Helsinki and according to Good Clinical Practice Guidelines.

### Statistical Analysis

Continuous variables are presented as means and standard deviations (SD) or as medians and interquartile ranges (IQR) as appropriate. Categorical data is presented as counts and proportions. Analysis was conducted according to the intention to treat principle. We compared between-group differences using T-test or Wilcoxon rank sum test for continuous data and using Χ^2^-statistics or Fisher’s exact test for categorical data as appropriate and report two-sided P values. We report mean changes and respective 95% confidence intervals (CIs) of outcome variables compared to baseline. Missing primary outcome data was inserted with the mean of the control group for analysis of the primary outcome in order to prevent bias from selective drop-out, as predefined in the study protocol. For the construction of the PACIC summary score one missing item out of 20 was allowed, other missing data was left as missing. When two eyes were affected by W-AMD in the same patient, every included eye in the study was analyzed separately. A two-sided alpha of 0.05 was set as level of significance for all comparisons. Statistical analyses were calculated using R Statistics version 3.1.0.

## Results

### Patient characteristics

**Fig 1:**
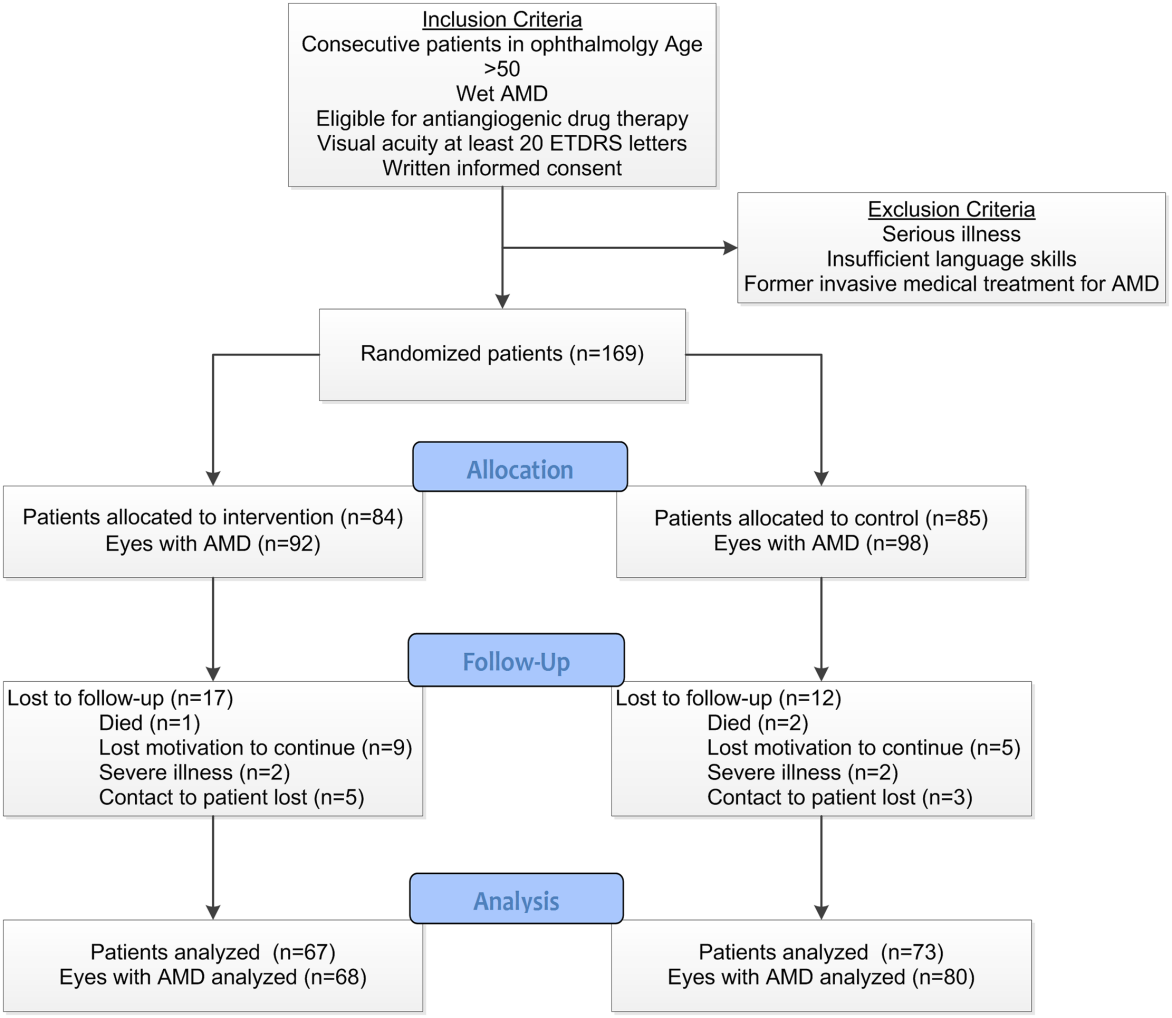
Flow Chart of the study. Fig 1 shows the flow chart of the study. The number of patients as well as the number of eyes with W-AMD is shown through the process.

Of 169 recruited patients, 84 were randomized to the intervention group, 85 to the control group. Eight patients in the intervention group and 13 patients in the control group had both eyes affected with W-AMD, thus, the intervention group comprised 92 eyes, the control group 98 eyes under the respective treatment protocols. The mean age of the patients was 76.7 (±8.0) years, 107 (63.3%) were female. 111 (65.7%) of the patients suffered from at least one other additional chronic condition. A detailed description of the study samples baseline characteristics and the recruiting study sites has been published and is openly available.[26] During the 12 month follow-up period, the drop-out rate was similar in both groups (17 dropouts [20.2%] in the intervention group and 12 [14.1%] in the control group), reasons for drop out did not differ significantly (Fig 1, Fisher’s exact test: p = 0.68). Table 1 displays the baseline characteristics, the groups appear to be well matched. Remarkably, patients in the control group, per chance, had a 4 letters lower median ETDRS score at baseline (Wilcoxon rank sum test: p = 0.16).

**Table 1:** Baseline characteristics of study population.

Variable (description method)	Intervention n = 84		Control n = 85	
Age in years (mean ±sd)	76.5	± 8.2	76.9	± 8.2
Female sex (n and %)	58	69.0%	49	57.6%
Education years (mean ±sd)	11.3	± 2.8	12.6	± 2.8*
Positive family history for wet AMD (n and %)	12	14.3%	16	18.8%
Diabetes (n and %)	8	9.5%	6	7.1%
Arterial hypertension (n and %)	42	50.0%	50	58.8%
Coronary heart disease(n and %)	12	14.3%	17	20.0%
Heart failure (n and %)	3	3.6%	5	5.9%
Cerebrovascular disease (n and %)	8	9.5%	6	7.1%
Chronic obstructive pulmonary disease (n and %)	7	8.3%	9	10.6%
Malignant disease (n and %)	5	6.0%	6	7.1%
Current or former smoker (n and %)	33	39.3%	42	49.4%
PHQ9 summary score^1^ (median and IQR)	2	1 to 4	2	1 to 4
ETDRS visual acuity of eyes with wet AMD^2^ (median and IQR)	62	47 to 73	58	43 to 73
OCT retinal thickness (median and IQR)	364	279 to 415	331	270 to 454
NEI-VFQ25^3^ composite score (median and IQR)	85	75 to 93	88	80 to 93
PACIC^4^ summary score (median and IQR)	2.4	1.8 to 3.2	2.4	1.7 to 3.2

^1^The PHQ9 is a measure of depression and its severity (range 0 to 27) as a self-administered questionnaire, values between 0 and 4 are interpreted as minimal depressive symptoms not requiring specific treatment

^2^The total n for this variable is the number of eyes, i.e. 92 in the intervention group and 98 in the control group

^3^The VFQ-25 composite score ranges from 0 to 100 and is a measure of visual function specific quality of life, 0 represents the lowest quality of life, 100 the highest quality of life.

^4^The PACIC summary score ranges from 1 to 5 and is a measure of the chronic illness care concordance to the chronic care model from the patient’s perspective, 1 represents the lowest concordance, 5 represents the highest concordance.

*Welch Two Sample t-test, p = <0.05

### Primary outcome

**Fig 2:**
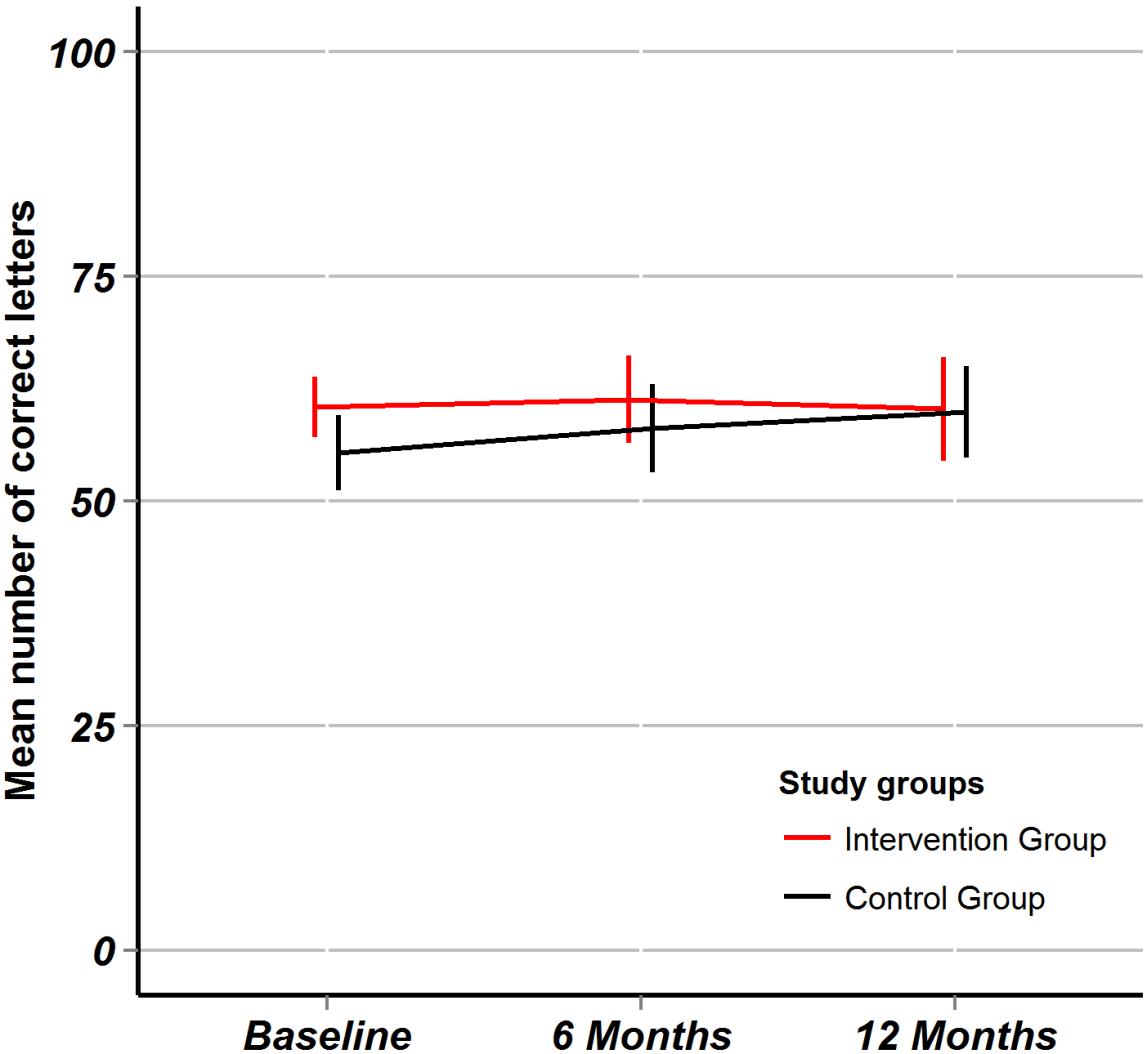
ETDRS visual acuity. Fig 2 shows outcome data for the ETDRS visual acuity through the study period. Point estimates are mean numbers of correctly identified letters, error bars represent 95% confidence intervals. The red line is for the intervention group, the black line is for the control group.

After 6 months, the mean change of ETDRS visual acuity in the intervention group was +0.3 (95% CI: -3.4 to +4.0) letters, in the control group +2.7 (95%CI: -1.0 to +6.4) letters, the between-group difference in mean change was -2.4 (95%CI: -7.6 to +2.8, T-test for equal mean differences: p = 0.36). After 12 months, the mean change of ETDRS visual acuity in the intervention group was -0.3 (95%CI: -4.4 to +3.8) letters, in the control group +4.5 (95%CI: +0.1 to +8.9) letters, the between-group difference in mean change was -4.8 (95%CI: -10.8 to +1.2, T-test for equal mean differences: p = 0.15). The targeted superiority of +5 ETDRS letter in favor of the intervention group remained unreached. The trend of mean ETDRS visual acuity over time is plotted (Fig 2).

### Secondary outcomes

**Fig 3:**
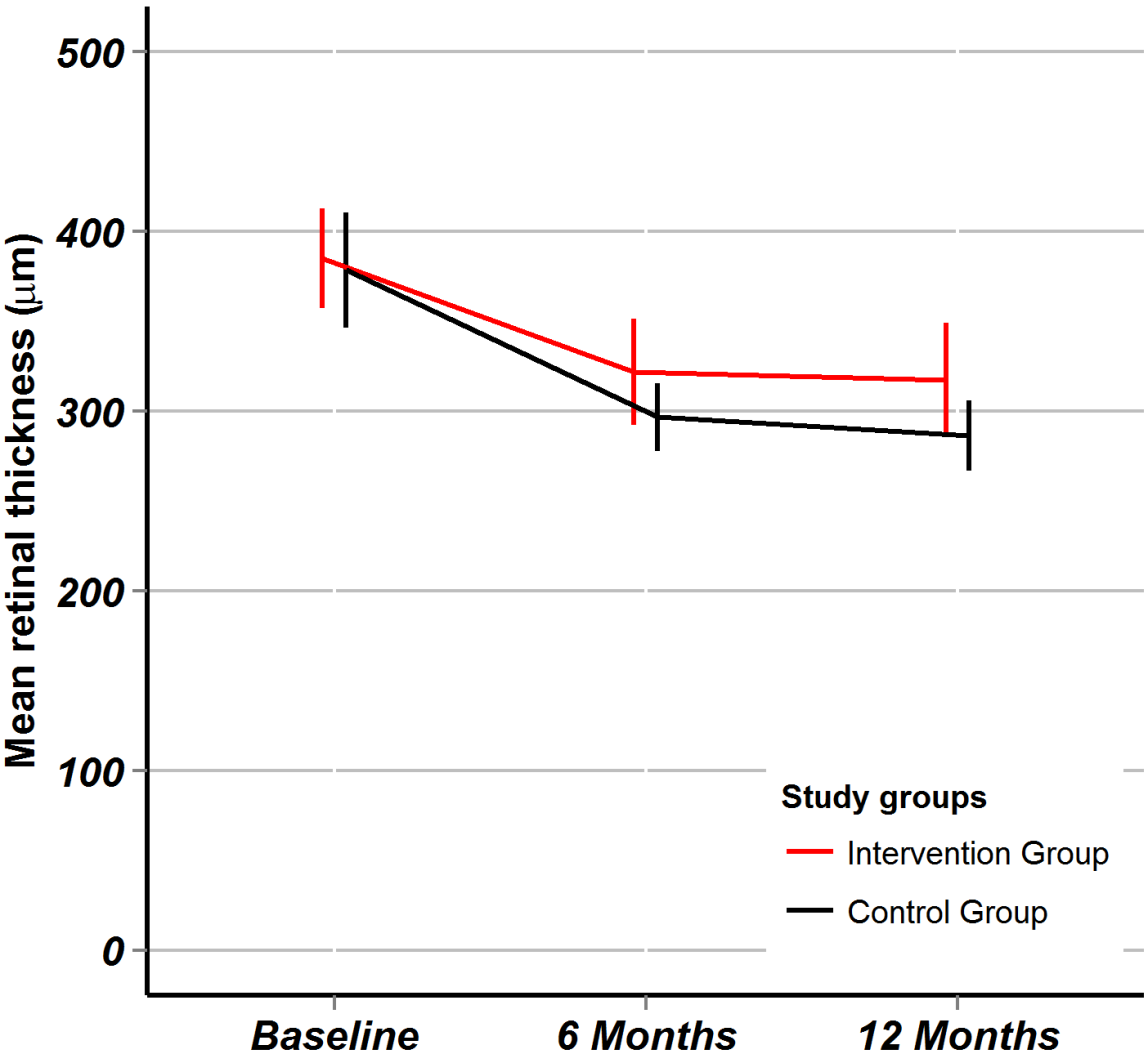
Optical coherence tomography retinal thickness. Fig 3 shows the optical coherence tomography (OCT) macular retinal thickness in micrometers. Point estimates are mean values, error bars represent 95% confidence intervals. The red line is for the intervention group, the black line is for the control group.

OCT retinal thickness: After 6 and 12 months there were significant improvements of OCT retinal thickness within both groups (Table 2), the between-group differences in mean change were, however, was not statistically significant: at 6 months +21.0 (95%CI: -30.4 to +72.3, p = 0.42) and at 12 months +14.0 (95%CI: -39.6 to +67.6, p = 0.60). The trend of retinal thickness over time is plotted (Fig 3).

**Table 2:** Within group mean changes and 95% confidence intervals in secondary outcomes.

Variable (description method)	Follow-up	Intervention group	Control group
OCT retinal thickness (μm)	6 months	-62.0 (95%CI: -96.3 to -27.7)	-82.9 (95%CI: -121.7 to -44.2)
OCT retinal thickness (μm)	12 months	-77.7 (95%CI: -119. to -35.6)	-91.7 (95%CI: -125.5 to -57.8)
NEI-VFQ25^1^ (composite score)	6 months	+2.1 (95%CI: -0.4 to +4.6)	+2.4 (95%CI: -0.3 to +5.1)
NEI-VFQ25^1^ (composite score)	12 months	+3.4 (95%CI: +1.1 to +5.7)	+1.3 (95%CI: -1.2 to +3.8)
PACIC^2^ (summary score)	12 months	+0.6 (95%CI: +0.1 to +1.0)	+0.6 (95%CI: +0.2 to +1.0)

^1^The VFQ-25 composite score ranges from 0 to 100 and is a measure of visual function specific quality of life, 0 represents the lowest quality of life, 100 the highest quality of life.

^2^The PACIC summary score ranges from 1 to 5 and is a measure of the chronic illness care concordance to the chronic care model from the patient’s perspective, 1 represents the lowest concordance, 5 represents the highest concordance.

**Fig 4:**
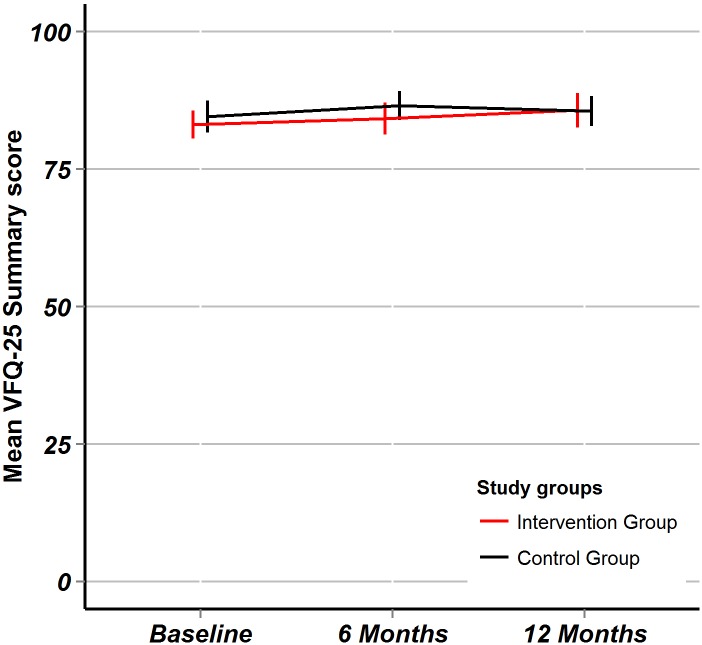
NEI VFQ-25 quality of life. Fig 4 shows the visual function specific quality of life measured by the NEIVFQ-25 composite score. Point estimates are mean values, error bars represent 95% confidence intervals. The red line is for the intervention group, the black line is for the control group.

NEI VFQ-25 summary score (reflecting the disease-specific quality of life): After 6 months, there was no significant change in NEI VFQ-25 summary score in either group, after 12 months the score improved significantly in the intervention group (Table 2). The between-group differences in mean change were, however, not statistically significant: at 6 months -0.3 (95%CI: -3.9 to +3.4, p = 0.87) and at 12 months +2.1 (95%CI:-1.3 to 5.5, p = 0.18). The trend of the NEI VFQ-25 summary score over time is plotted (Fig 4).

PACIC summary score (patient perceived accordance of care to the CCM): After 12 months, the PACIC summary score improved significantly in both groups (Table 2), however, without any between-group difference: 0.0 (95%CI: -0.6 to +0.6, p = 0.99).

**Fig 5:**
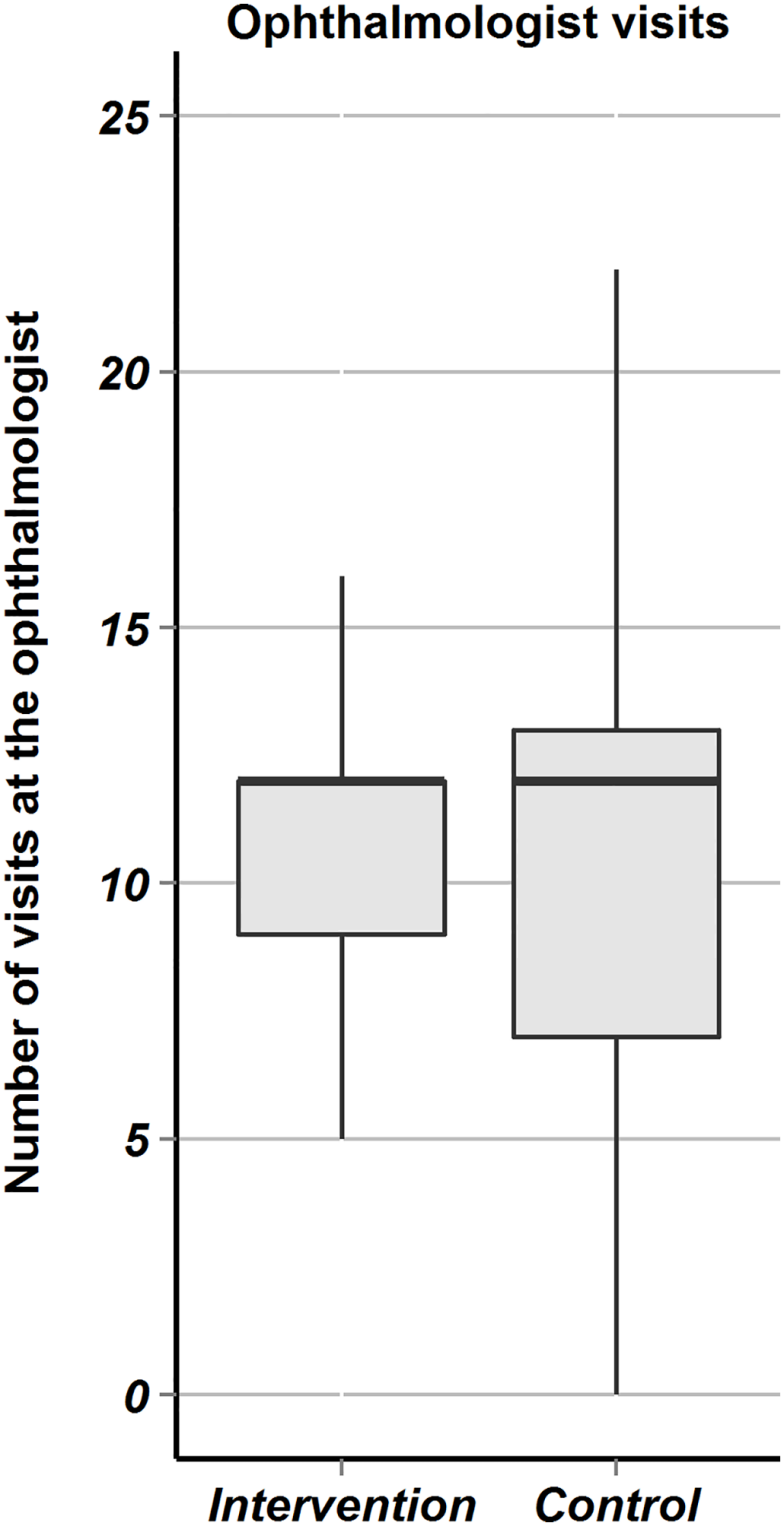
Clinic visits at the ophthalmologists. Fig 5 are boxplots comparing the intervention versus the control groups with regard to the number of visits at the ophthalmologist because of W-AMD. The hinges are the first and third quartile, the horizontal line is the median, the whiskers extend from the hinges to the value within 1.5 times the IQR.

After 12 months, the median number of ophthalmologist visits because of W-AMD in the intervention group was 12 (IQR: 9 to 12) and in the control group also 12 (IQR: 7 to 13, Wilcoxon rank sum test: p = 0.523). There was clearly greater variability in follow-up in the control group (Fig 5) arguing that care in the intervention group was more structured, however, without resulting in improved outcomes as demonstrated above. Concerning family physician consultations and hospitalizations no significant differences between the study groups appeared (data not shown).

The mean ACIC score which measures the study site perceived accordance of care to the CCM at baseline was 5.6 (±2.18). After 12 months the trend went upwards, the mean change, however, was not statistically significant: +1.2 (95%CI: -0.6 to +2.9, paired Wilcoxon rank sum test: p = 0.09).

## Discussion

In this randomized controlled study we tested the effect of core elements of the CCM on relevant outcomes in W-AMD. We found no significant effect of the CCM intervention for any of the assessed outcomes. Moreover, the between-group difference in mean change of the visual acuity failed not only to achieve statistical significance but the targeted superiority of +5 ETDRS letter was not even reached by the upper border of the 95% confidence interval. In view of the distribution of these results, we see our hypothesis of superiority of the CCM strongly antagonized even without reaching the intended sample size in this trial. We must therefore acknowledge that the implementation of core elements of the CCM was ineffective to improve the most important outcomes in AMD.

Beneficial results of the CCM found in many other chronic conditions were not reproducible in AMD. For this finding, several explanations are possible because W-AMD might differ importantly from classic chronic conditions. Regarding for instance the natural course of W-AMD, phases of disease activity and inactivity alternate and so far, no modifiable factors that could be targeted by chronic care interventions are known. This is very much in contrast to most classical chronic conditions, where modifiable risk factors are very well established and targeting those is clearly effective. In W-AMD, our intervention focused mainly on the early detection of disease activity introducing self-monitoring. Self-monitoring itself has clear advantages in many chronic conditions when an otherwise undetectable measure of disease activity is monitored (such as blood sugar in diabetes or blood pressure in hypertension). Again generalizability from classic chronic conditions to W-AMD might be limited because also naïve patients might detect disease activity with great sensitivity when noticing visual loss, in contrast to a diabetic for example that very much depends on empowerment in self-monitoring to detect hyperglycemia. Self-monitoring is already occurring naturally in daily living in AMD, thus the effects of an additional self-monitoring support might be much smaller than in most chronic conditions and a simple Amsler Test might not bring decisive advantages. Similarly, benefits from empowerment of patients with decision aids and action plans might be limited in AMD. Generally, patients benefit from decision aids or action plans, when a multitude of options is available within a complex framework where several circumstances need to be considered. Again, diabetes is a good example for CCM effectiveness: The activated and informed patient knows how to counteract hyperglycemia following sophisticated action plans covering physical activity, nutritional changes and even autonomous adaptation of pharmacotherapy. In W-AMD however, patients noticing a relevant deterioration have very little options: call the ophthalmologist. Patients with W-AMD are very well aware of this option and the introduction of decision aids and action plans might yield only minimal additional benefit.

Interestingly, in our study we found that patients with W-AMD had importantly better visual functioning at baseline than in previous studies with similar inclusion criteria or real life observational studies: Whereas we expected to include patients with an average ETDRS visual acuity around 48 to 55 letters, the patients in our study had a mean ETDRS visual acuity of 58 letters.[4–7, 24, 25] This finding can be explained by the setting of the study. Most patients were recruited in urban regions in hospital run ophthalmology clinics. Arguably, these were patients with a very good access to healthcare in an already comparably wealthy healthcare system. These circumstances might have further limited the additional effects of the CCM implementation of our study because also usual care for those patients was already highly organized and featured many characteristics of the CCM. This is reflected by the comparably high ACIC scores we found in the trial and by the non-differing and equally risen PACIC scores of the study groups.

This study has strengths and limitations. It is the first study evaluating effects of core elements of the CCM on patients with W-AMD, a disease where treatment protocols are still to debate and a relevant evidence-performance gap exists. The most important limitation of the study is that recruitment was more difficult than anticipated and the study was stopped early. The failure of the CCM implementation was, however, very clear, even if the intended sample size was not reached. We are confident, that more patients would not have completely reversed the trend that appeared to be even in favor of the control group. Another limitation was the open label design of the study, the awareness of allocation in the intervention group, however, would have introduced bias in favor of the intervention, our opposite finding rather in favor of the control group is thus added credibility to our rejection of the study hypothesis. However, as we randomized on the patient level, contamination bias may have occurred: Even if the groups were managed separately in the study sites and procedures were monitored, some aspects of the CCM might unintentionally have influenced the performance in the control group. The most important aspects of the intervention (structured follow-up phone calls and Amsler test self-measurement instructions), however, were definitely only accessible for the intervention group. It is, however, also safe to assume that “usual care” in the control group also featured some form of structured follow-up in the highly performing Swiss healthcare system. The intervention might thus not have been strong enough to achieve an important difference in a health system with an already successful follow-up as reflected by the non-different median number of 12 ophthalmologist visits also found in the control group. Possibly a longer follow-up time than 12 months would have been required to show beneficial effects of the CCM implementation. Ultimately, we did not assess the intended treatment protocol of each individual patient (fixed monthly injections, PRN or treat and extend), nor did we assess the exact number of injections. In our framework however, these were only the mediators of the outcomes that should have been enhanced by the CCM implementation. Successful follow-up is crucial under all treatment protocols and naturally associated with the number of injections. Improving-follow up should thus improve outcomes regardless of the individual treatment protocol.

Concluding, we must state that our intervention aiming at improving chronic care was not associated with favorable outcomes within 12 months in W-AMD. Other approaches need to be tested to close the evidence-performance gap in W-AMD.

## Supporting Information

S1 CONSORT Checklist CONSORT Checklist. (DOC)

S1 Protocol Version accepted by ethics committee. (DOCX)
